# Effect of Process Parameters on the Appearance of Defects of Flake-Pigmented Metallic Polymer

**DOI:** 10.3390/polym16152193

**Published:** 2024-08-01

**Authors:** Seungkwon Choi, Naksoo Kim

**Affiliations:** Department of Mechanical Engineering, Sogang University, Seoul 04107, Republic of Korea; csg7669@sogang.ac.kr

**Keywords:** metallic polymer, injection molding, appearance defect, flake pigment, fiber orientation tensor, process parameters

## Abstract

This study investigates the influence of the main process parameters of injection molding(mold temperature, melt temperature, and injection rate) on the appearance of defects of flake-pigmented metallic polymer parts. To understand the influence of process parameters, an appearance defects index (ADI) is proposed to quantify the appearance defects. In this process, we propose a criterion for judging the appearance of defects based on the results of fiber orientation and tensor distribution analyses of the skin layer, which is then verified analytically by simulating experiments from the literature. Using the Taguchi experimental method, we designed an $L_{25}$ orthogonal array to systematically evaluate the influence of process parameters. For each experimental condition, the signal-to-noise ratio (S/N ratio) was calculated to determine the optimal level of each factor and its influence on the appearance of defects. According to the results, mold temperature has the greatest influence on the appearance of defects, with an influence of 48.7%, followed by injection rate with an influence of 40.8%, and melt temperature with an influence of 10.5%. The optimal process parameters were found to be a mold temperature of 40 °C, a melt temperature of 250 °C, and an injection rate of 10 cm^3^/s, which resulted in a 12.6% improvement in the Appearance defects index (ADI) compared to the standard injection molding condition of ABS materials. This study confirmed that it is possible to improve the appearance of defects by adjusting the process parameters of injection molding.

## 1. Introduction

Metallic injection is a technology that produces parts with a metallic luster and reflective effects. It achieves this directly through injection molding by mixing metallic pigments, such as aluminum pigments, into polymers. This method is applied to home appliances, automotive interiors, cell phone cases, etc., providing a high-end appearance without additional surface treatment, enhancing the visual appeal of the product, and adding value. Compared to conventional post-treatment processes such as plating or painting, it is economically and environmentally friendly and is gaining attention for its advantages in simplifying the manufacturing process and eliminating additional surface finishing processes [1,2].

**Figure 1:**
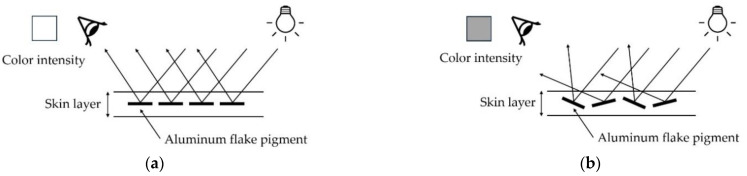

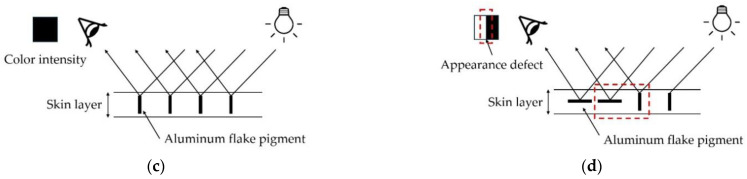
Surface appearance of reflection according to the flake orientation. (**a**) Parallel orientation (**b**) Irregular orientation (**c**) Perpendicular orientation (**d**) Appearance of defects.

However, the appearance of defects caused by flake pigments during metallic injection remains a persistent problem. The main cause of these defects is the irregular orientation and distribution of flakes, which mainly occur in the skin layer, as shown in Figure 1. During the injection molding process, as the molten polymer flows along the mold wall, reflective particles such as aluminum pigments are oriented parallel to the surface of the part, resulting in a uniform gloss with bright color intensity, as shown in Figure 1a. On the other hand, when the two different flows recombine behind obstacle structures such as ribs or holes, the flake pigments are tilted and oriented irregularly or perpendicularly, as shown in Figure 1b,c, which degrades the reflective properties of the surface and darkens the color intensity. As shown in Figure 1d, in areas where the orientation difference of the flake pigment is severe, the difference in color intensity causes a line-like appearance defect. Therefore, it is important that the flake orientation of the skin layer is aligned parallel to the surface to resolve the appearance of defects in metallic injection [2,3,4,5,6].

To solve this defects problem, previous studies have mainly focused on the content, size, and shape of flakes. Lim Jung Seop found that higher flake content reduces the appearance of defects by increasing the pigment concentration in the defect area, and adding inorganic fillers improves the orientation and distribution of flakes. In the flow line region, the lamellar flakes were arranged in random directions, while the 3D flakes showed a distinct and stable orientation. This suggests that using 3D flakes instead of lamellar flakes can improve the appearance of defects [7,8]. X Hong studied the effect of the size and distribution of aluminum flake pigments on the surface properties of HDPE/Al composites and found that the surface roughness increases with increasing pigment size, which reduces the metallic effect and reflectivity [9]. Nils Maximilian Demski studied the effect of tetrahedral pigments on metallic injection and found that the randomly distributed orientation of the tetrahedral pigment particles provides uniform optical properties, which helps to improve the appearance of defects [10]. According to references [11,12], for flake pigments, the larger surface area reflects the incident light as a whole, but the metallization effect decreases with decreasing particle size due to scattering at the edges. Additionally, spherical and rounded particles have a smaller reflective area and therefore reflect less light, reducing the metallic effect. Arfat Anis reported that the arrangement of flakes is determined by the direction of flow during the molding process, and flakes with a high aspect ratio are well arranged during this process, resulting in a visually less defective appearance. Furthermore, the strong adhesion between aluminum flakes and PET prevents the aluminum flakes from dislodging in the event of product breakage, which also contributes to reducing the appearance of defects [13].

However, very few studies have been conducted to improve the appearance of defects of metallic injection by controlling injection process parameters. In injection parts with glass or carbon fiber reinforcement, injection process parameters have a significant effect on fiber orientation [14,15,16], so it was assumed that injection process parameters would affect the appearance quality of metallic injection, where flake orientation is also important [3]. This study aims to determine the influence of injection process parameters on the appearance defects of metallic injection parts and optimize the process parameters using Taguchi’s experimental method. This study confirms that the appearance of defects can be improved by adjusting the injection process parameters and proposes a method to quantify the appearance of defects using the flake orientation distribution of the skin layer.

## 2. Materials and Methods

As discussed in the introduction, the appearance of defects in metallic injection is influenced by the flake orientation of the skin layer. In this study, we aim to analyze this flake orientation distribution to determine the appearance of defects. To do this, it was necessary to determine which flake orientation distribution causes the appearance of defects. To determine the criteria for judging the appearance of defects based on the flake orientation distribution, we performed analytical verification based on the experimental results from references [3,17]. Hereafter, we refer to the analysis that simulates the experiment in reference [3] as Case 1 and the analysis that simulates the experiment in reference [17] as Case 2.

### 2.1. Analytical Method for Determining Appearance of Defects

#### 2.1.1. Theory

This section describes the Folgar-Tucker model used to analyze flake orientation. The Folgar-Tucker model [18] is a widely used numerical approach to predict fiber orientation during injection molding. In Equation (1), w is the vorticity tensor, $\dot{\gamma }$ is the strain rate tensor, a is the fiber orientation tensor, $C_{l}$ is the interaction coefficient, and λ is the orientation factor [18]. Folgar and Tucker developed this model based on Jeffery’s equations, adding an isotropic rotational diffusion to account for interactions between fibers. The model calculates how fibers move and deform under hydrodynamic flow conditions, including w and $\dot{\gamma }$. $C_{l}$ and λ to effectively model the interaction between fibers and the effect of fiber geometry on orientation [19,20]. In this paper, the analysis was performed using the Moldflow program, and the flake orientation tensor (F.O.T) was calculated using the Folgar-Tucker model.

(1) $$\frac{Da_{ij}}{Dt}=-\frac{1}{2}\left(w_{ik}a_{kj}-a_{ik}w_{kj}\right)+\frac{1}{2}\lambda \left({\dot{\gamma }}_{ik}a_{kj}+a_{ik}{\dot{\gamma }}_{kj}-2a_{ijkl}\right)+2C_{l}\dot{\gamma }\left(\delta_{ij}-3a_{ij}\right)$$

#### 2.1.2. Validation Experiment

##### Material

The injection analysis was performed using the polypropylene (J106G, Prime Polymer Corporation, Japan, Tokyo) used in the reference [3], and aluminum flakes (NME060T4, Toyo Aluminum K.K, Japan) with an average diameter of 60 μm and thickness of 0.6 μm were added at 3 wt%. The injection molding conditions were set as follows: mold temperature of 40 °C, melt temperature of 210 °C, and injection rate of 30 cm^3^/s.

For Case 2, the injection analysis was performed using the ABS (XR-401, LG Chemical, Republic of Korea) used in reference [17], with an average diameter of 13 μm and thickness of 1 μm, and aluminum flake (Silberline, USA, Tamaqua) added at 0.5 wt%. The injection molding conditions were set as follows: mold temperature of 60 °C, melt temperature of 240 °C, and injection rate of 38 cm^3^/s.

##### Specimen

**Figure 2:**
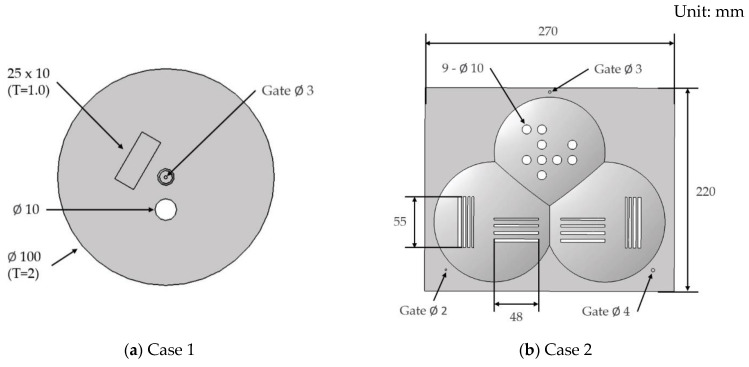
Specimen geometry. (**a**) Case 1 (**b**) Case 2.

To observe the orientation of the aluminum flake and the resulting surface appearance, the modeled specimen geometries are shown in Figure 2. Case 1 has an overall size of 100 mm in diameter and an average thickness of 2 mm, while Case 2 has an overall size of 270 × 220 mm and an average thickness of 1.8 mm.

#### 2.1.3. Validation Experiment Result

The process parameters for the injection analysis used the injection molding conditions from Material Section in Section 2.1.2. The mesh type was 3D mesh with 226,453 elements (size 0.3 mm) for Case 1 and 934,755 elements (size 0.5 mm) for Case 2.

**Figure 3:**
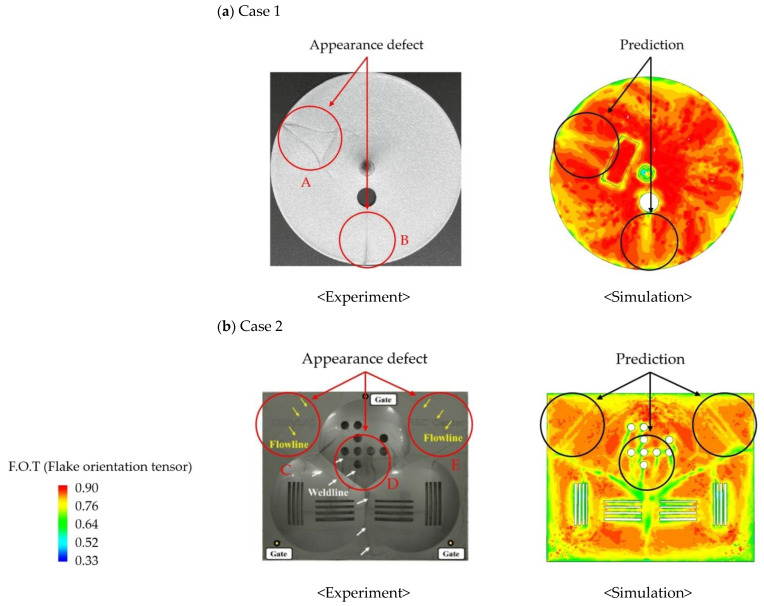
The result of the experiment and simulated distribution of flake orientation tensor (F.O.T). (**a**) Case 1 (**b**) Case 2., A–E were regions where flake orientation differences occurred.

**Figure 4:**
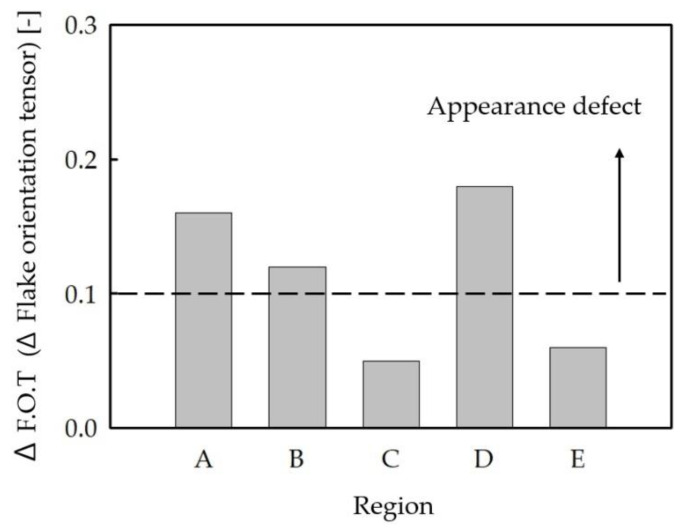
Differences in flake orientation tensor (F.O.T) between regions A to E. Regions A and B are results from case 1, and Regions C to E are results from case 2.

After analyzing Cases 1 and 2, the regions where the flake orientation degraded sharply compared to the surrounding area tended to coincide with the actual appearance defects, as shown in Figure 3. In Case 1, the regions A and B, where the flake orientation degrades sharply compared to the periphery due to the obstacle structure, were observed to have weld line-shaped defects as shown in the experimental results. Similarly, in Case 2, weld line-shaped defects were observed in region D, where the flake orientation degraded rapidly, and flow line-shaped appearance defects occurred in regions C and E. In addition, more severe appearance defects occurred when the difference between the flake orientation of the flake prediction part and the periphery was greater than 0.1, such as in regions A, B, and D. In regions C and E, where the flake orientation difference was 0.1, relatively small appearance defects occurred compared to regions A, B, and D (see Figure 4).

The above results confirm that in areas where the flake orientation difference is large, the flake orientation is not aligned with the flow, resulting in a difference in reflectivity, which ultimately causes the appearance defects. The experimental results in reference [5], where the flake orientation difference causes a difference in reflectivity between the product surface and the flake through the analysis of SEM images of the appearance defects, support these results. However, in Figure 3, in addition to Regions A~E, there were regions where flake orientation differences occurred, but the appearance defects were not obvious. This is either because the appearance defects are overlapped with the fillet shape and are not visible, or because the flake orientation difference changes gradually rather than abruptly, resulting in a very small appearance defect.

In this study, we ignore the appearance defects that do not critically affect the appearance quality, such as flow lines in regions C and E, and focus on the appearance defects that critically affect the appearance quality, such as weld lines in regions A, B, and D. Based on the results of the flake orientation distribution analysis in Section 2.1, it was found that appearance defects occur in the following two cases:

- Appearance defects occur in regions where the flake orientation difference (ΔF.O.T.) degrades rapidly by more than 0.1.
- The larger the flake orientation difference (ΔF.O.T.), the worse the appearance of defects.

### 2.2. Effect of Processing Parameter on Appearance Defects

#### 2.2.1. Quantification of Appearance Defects

**Figure 5:**
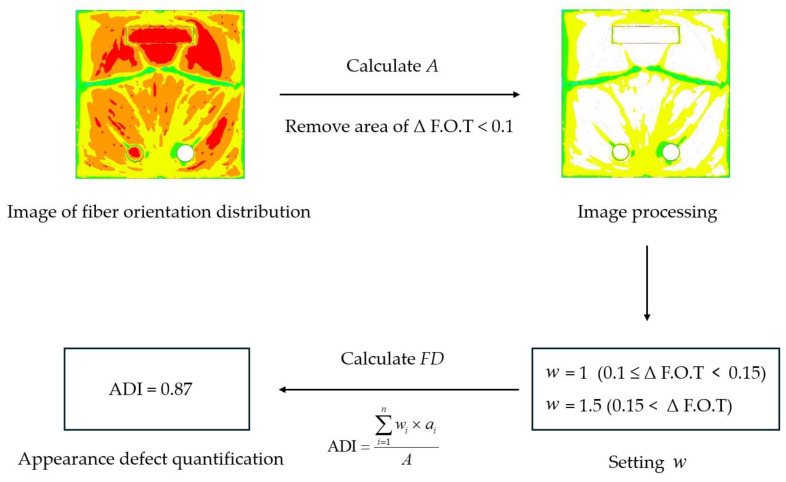
Process of Appearance defect quantification.

To analyze the correlation between injection process parameters and appearance defects, a method to quantify appearance defects is needed. In this study, we propose a method to quantify the appearance defects by introducing the appearance defect index (ADI) as shown in Figure 5. The appearance defects index (ADI) can be expressed as shown in Equation (2), where w is the weight factor according to the flake orientation difference, A is the total area of the product, a is the unit area of the appearance defect by converting the area of each pixel in the appearance defect to the actual area, and n is the total pixel count of the appearance defect. The quantitative method for appearance defects is as follows:

1. Extract an image of the flake orientation distribution through injection analysis.
2. Calculate the total pixel area in the image with a flake orientation distribution and convert it to the actual area (A). (The actual area calculated here is the total area of the product).
3. Remove the remaining pixels, keeping only those in areas where the flake orientation difference drops sharply by more than 0.1 (use RGB values to remove pixels).
4. Set weight factor (w) based on flake orientation differences.
5. Calculate the remaining pixel area based on the weight factor you set w and convert it to the actual area to calculate a. (The remaining pixel area is the total area of the product with the appearance defects)
6. Calculate ADI using expression (2).

First, we extract the flake orientation distribution image by calculating the flake orientation tensor using a commercial program such as Moldflow and then calculate the area of the entire product (A) using the pixel area. Then utilizing the results from Section 2.1.3, remove the pixels in the area where the flake orientation difference is more than 0.1, remaining only the pixels in the area where the flake orientation difference drops sharply. The larger the orientation difference, the more severe the appearance defect, so we set a weight factor (w) based on the range of the orientation difference. Finally, the remaining pixel area is converted to the actual area according to the weight factor (w) to calculate a and get the Appearance Defect Index (ADI). The weights can be set by the user based on the importance of the area and severity of the appearance defect. For example, if the area of the appearance defect is important, w can be set close to 1, and if the severity of the appearance defect is important, it can be set close to 1.5, allowing for adjustments according to the objective. In this study, when the flake orientation difference is 0.1 ≤ ΔF.O.T < 0.15, w is set to 1, and when 0.15 ≤ ΔF.O.T, w is set to 1.5.

(2) $$\mathrm{ADI}(\mathrm{Appearance}\;\;\mathrm{defect}\;\;\;\;\mathrm{index})=\frac{\sum_{i=1}^{n}w_{i}\times \;\;a_{i}}{A}$$

#### 2.2.2. Material

ABS (XR-401, LG Chemical, Republic of Korea) was used as the material, and aluminum flake (NME060T4, Toyo Aluminum K.K., Japan) with an average diameter of 60 μm and a thickness of 0.6 μm was added at 3 wt%. Under the standard injection molding condition (Table 1), the appearance defect index (ADI) was analyzed when the values of the three main parameters of the injection molding process, mold temperature, melt temperature, and injection rate, were changed and the results are summarized in Section 3.1.

#### 2.2.3. Specimen

**Figure 6:**
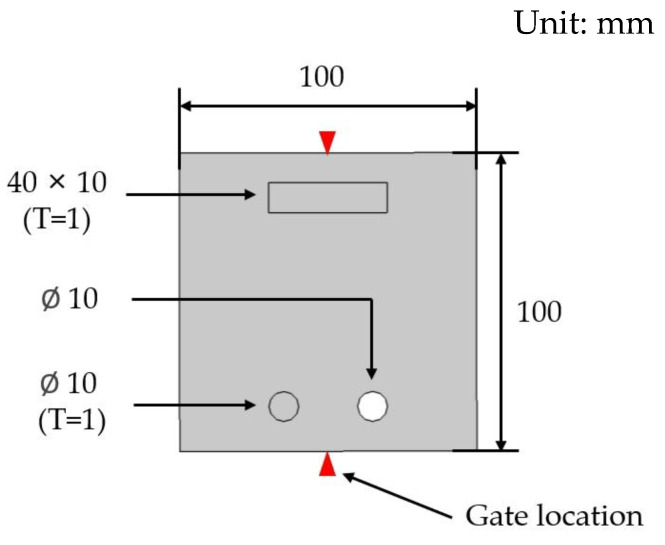
Specimen geometry.

The specimen geometry modeled to analyze the correlation between injection process parameters and appearance defects is shown in Figure 6. One square and two circular obstacles were included to induce appearance defects, with an overall size of 100 × 100 mm and an average thickness of 2 mm.

#### 2.2.4. Optimization of Injection Molding Process Parameters

In this study, Taguchi’s experimental method (3 factors, 5 levels) is used to evaluate the importance of the injection process parameters and to derive the injection process parameters with the minimum ADI. The factors use the same process parameters as in Section 2.2.1, and the ranges are as follows: A (mold temperature) is set to 40~80 °C, B (melt temperature) is set to 230~270 °C, and C (injection rate) is set to 5~25 ${cm}^{3}/s$ (Table 2). The objective function is set to the appearance defect index (ADI), which quantifies the appearance defect, and a total of 25 experiments are conducted. The signal-to-noise ratio (S/N) of each experiment is calculated to determine the influence of the process parameters, and the optimal process parameter that minimizes the ADI is found. S/N is defined as shown in Equation (3), where $Y_{i}$ is the ADI value for each experimental condition and n is the number of experiments. The experiments used $L_{25}$ Taguchi’s orthogonal array is shown in Table 3.

(3) $$S/N=10\log\left[\frac{1}{n}\sum_{i=1}^{n}Y_{i}^{2}\right]$$

## 3. Results and Discussion

### 3.1. Effect of Process Parameters on Appearance Defects

**Figure 7:**
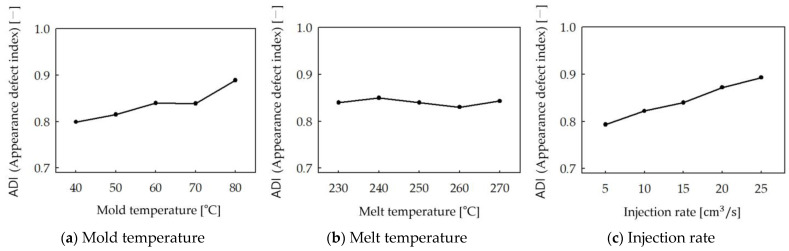
The effect of process parameters on ADI. (**a**) Mold temperature (**b**) Melt temperature (**c**) Injection rate.

**Figure 8:**
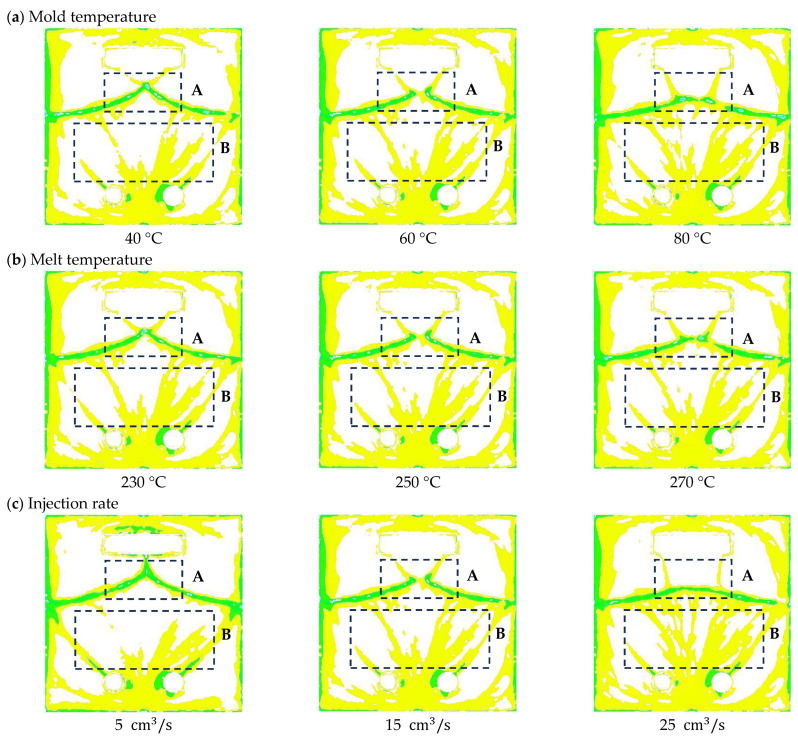
Result of image processing. (**a**) Mold temperature at 40 °C, 60 °C, 80 °C (**b**) Melt temperature at 230 °C, 250 °C, 270 °C (**c**) Injection rate at 5 ${cm}^{3}/s$, 15 ${cm}^{3}/s$, 25 ${cm}^{3}/s$., A and B were regions where appearance defect occurred.

Figure 7 and Figure 8 show the results of analyzing the Appearance Defect Index (ADI) when the process parameter values change according to the process of appearance defect quantification presented in Section 2.2.1. Figure 8 is the result of image processing of appearance defects. Yellow areas indicate appearance defects with a flake orientation difference of more than 0.1 but less than 0.15, and green areas indicate appearance defects with a flake orientation difference of more than 0.15. Figure 7 is a graph showing the correlation between the process parameter values and the Appearance Defect Index (ADI). The analysis results are as follows:

#### 3.1.1. Mold Temperature

To simulate the effect of mold temperature, standard molding conditions (Table 2) were used, and the mold temperature was varied to 40, 50, 60, 70, and 80 °C. As shown in Figure 8a, as the mold temperature increases, the change in appearance defects is largest in region A, the area where the two flows merge due to the square obstacle. The area of green defects decreases, but the area of yellow defects increases, and the area of yellow defects also increases in Region B. In Figure 7a, the ADI value tends to increase as the mold temperature increases.

#### 3.1.2. Melt Temperature

To simulate the effect of melt temperature, standard molding conditions (Table 2) were used, and the melt temperature was set to 230, 240, 250, 260, and 270 °C. In Figure 8b, the change in appearance defects in region A is the largest as the melt temperature increases, but the change is small compared to the mold temperature. In region B, the change in appearance defects is small, and the change in ADI value tends to be insignificant as the melt temperature increases in Figure 7b.

#### 3.1.3. Injection Rate

To simulate the effect of injection rate, standard molding conditions (Table 2) were used, and the injection rate was varied to 5, 10, 15, 20, and 25 cm³/s. In Figure 8c, the variation in region A was similar to that with the mold temperature. Region B also showed similar results to the mold temperature, and it can be seen from Figure 7c that the ADI value tends to increase as the injection rate increases.

### 3.2. Taguchi’s Experimental Method

**Figure 9:**
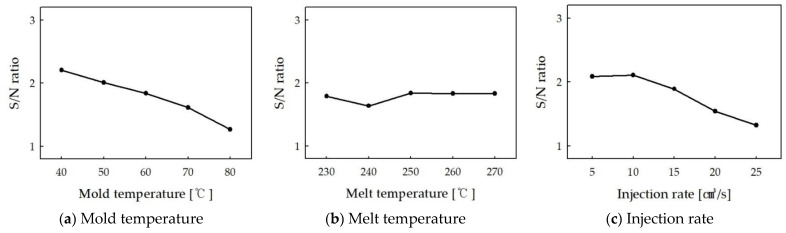
S/N ratio of process parameters. (**a**) Mold temperature (**b**) Melt temperature (**c**) Injection rate.

The average S/N ratios for each process parameter level are shown in Table 4, and these ratios were calculated based on the experimental data obtained from the orthogonal array table. Mold temperature was found to be the most influential factor in the appearance of defects, with an influence of 48.7%. As can be seen in Table 4, the S/N ratio decreases as the mold temperature increases from 40 °C to 100 °C. This indicates that lower mold temperature tends to reduce the appearance of defects. At higher mold temperatures, the melt cools rapidly, resulting in rapid stress relaxation. However, asymmetric stress relaxation during this process results in an uneven arrangement of flake pigments, which manifests as appearance defects [21]. The optimal mold temperature was 40 °C, and the average S/N ratio at this level was the highest at 2.21. Melt temperature had the least effect on appearance defects of the three factors, with an influence of 10.5%. The S/N ratio for different melt temperature levels showed relatively consistent values, with only slight variations. The optimal melt temperature was 250 °C, with an average S/N ratio of 1.84 at this level. Injection rate was the second most influential factor, with a 40.8% impact on appearance defects. The S/N ratio was highest at an injection rate of 10 cm³/s, with a value of 2.11. At higher injection rates, the S/N ratio decreased, indicating that higher injection rates tend to increase appearance defects. At higher mold temperatures, the melt cools rapidly, resulting in rapid stress relaxation. However, asymmetric stress relaxation during this process results in an uneven arrangement of flake pigments, which manifests as appearance defects [22]. The trend of the S/N ratio is plotted in Figure 9 for easy understanding.

Based on the S/N ratio analysis, the optimal process parameters to minimize appearance defects are:

- Mold temperature: 40 °C;
- Melt temperature: 250 °C;
- Injection rate: 10 cm^3^/s.

**Figure 10:**
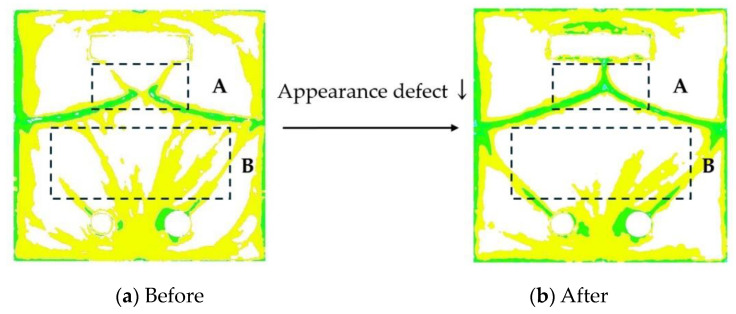
Result of image processing. (**a**) Before (**b**) After.

Using Taguchi’s experimental method to compare the ADI results of the optimized process parameters and the default recommended process parameters, the results show that the ADI value of the optimized process parameters is improved by about 12.6% compared to the default recommended process parameters (See Table 5). The image processing of appearance defects with process parameters before and after optimization is shown in Figure 10, and the appearance defect area in regions A and B is reduced in the optimized process parameter result. This indicates that the appearance of defects can be improved by adjusting the process parameters.

## 4. Conclusions

In this study, we investigated the impact of injection process parameters on appearance defects in metallic injection molded parts and came to three conclusions:

1. The flake orientation distribution of the skin layer can be used to judge the appearance quality and has the following two characteristics. The first is that appearance defects mainly occur in areas where the flake orientation difference is more than 0.1, and the second is that the larger the flake orientation difference, the worse the appearance defects. Based on these features, this study proposes an appearance defect index (ADI) that can quantitatively express appearance defects.
2. The results of the trend of the Appearance Defect Index (ADI) according to the injection process parameters are as follows: the ADI value tends to increase as the mold temperature and injection rate increase, but the change in ADI value tends to be insignificant as the melt temperature increases.
3. Checked the effect of injection process parameters on appearance defects for ABS material and found that the order of influence is mold temperature (48.7%) > injection speed (40.8%) > melt temperature (10.5%). Also, optimized the process parameters using Taguchi, and found that the lowest ADI value was obtained when the mold temperature was 40 °C, the melt temperature was 250 °C, and the injection speed was 10 cm^3^/s. This result was 12.6% better than before.

The above conclusions confirm that injection process parameters have a significant impact on the appearance defects of in metallic injection parts. However, this study is limited by the fact that the ADI of localized areas is not available to calculate the ADI of the whole part. In the future, it will be necessary to minimize the ADI by specifying the local areas where the appearance defects should be improved. In this process, it is necessary to improve the appearance of defects by optimizing the injection process parameters using various optimization techniques and to verify the improvement effect experimentally.

## Figures and Tables

**Table 1:** Standard injection molding condition.

Parameter	Value
Mold temperature	60
Melt temperature	250
Injection rate	15 ${cm}^{3}/s$

**Table 2:** Factor and level of $L_{25}$ Taguchi’s experimental method.

Factor	Name	Levels				
		1	2	3	4	5
A	Mold temperature [°C]	40	50	60	70	80
B	Melt temperature [°C]	230	240	250	260	270
C	Injection rate [${cm}^{3}/s$]	5	10	15	20	25

**Table 3:** $L_{25}$ Taguchi’s orthogonal array.

No.	A	B	C	ADI [−]
1	40	230	5	0.734
2	40	240	10	0.758
3	40	250	15	0.762
4	40	260	20	0.795
5	40	270	25	0.833
6	50	230	10	0.770
7	50	240	15	0.784
8	50	250	20	0.822
9	50	260	25	0.837
10	50	270	5	0.757
11	60	230	15	0.804
12	60	240	20	0.846
13	60	250	25	0.855
14	60	260	5	0.766
15	60	270	10	0.778
16	70	230	20	0.870
17	70	240	25	0.868
18	70	250	5	0.791
19	70	260	10	0.801
20	70	270	15	0.825
21	80	230	25	0.903
22	80	240	5	0.893
23	80	250	10	0.823
24	80	260	15	0.849
25	80	270	20	0.856

**Table 4:** Response value of S/N Ratio FD.

Factors	Levels (Mean of S/N Ratio)					Contribution [%]	Rank
	1	2	3	4	5		
A	2.21	2.01	1.84	1.62	1.27	48.7	1
B	1.79	1.64	1.84	1.83	1.83	10.5	3
C	2.09	2.11	1.89	1.54	1.32	40.8	2

**Table 5:** ADI results before and after optimization.

	Mold Temperature [°C]	Melt Temperature [°C]	Injection Rate $\left[{cm}^{3}/s\right]$	ADI [-]
Before	60	250	15	0.831
After	40	250	10	0.726

## Data Availability

The original contributions presented in the study are included in the article, further inquiries can be directed to the corresponding author.
